# Serial Galactose-Deficient IgA1 Levels in Children with IgA Nephropathy and Healthy Controls

**DOI:** 10.1155/2017/8210641

**Published:** 2017-11-26

**Authors:** John T. Sanders, M. Colleen Hastings, Zina Moldoveanu, Jan Novak, Bruce A. Julian, Zoran Bursac, Robert J. Wyatt

**Affiliations:** ^1^Sanford Children's Hospital, Sioux Falls, SD 57117, USA; ^2^University of Tennessee Health Sciences Center, Memphis, TN 38013, USA; ^3^Children's Foundation Research Institute, Memphis, TN 38013, USA; ^4^University of Alabama at Birmingham, Birmingham, AL 35294, USA

## Abstract

Galactose-deficient IgA1 (Gd-IgA1) is a key pathogenic factor for IgA nephropathy (IgAN) and a potential biomarker for the disease. This study examined serial serum Gd-IgA1 levels over 1 year in 13 children with IgAN and 40 healthy children, to determine whether or not serum Gd-IgA1 levels changed over time. Subjects were younger than 18 years of age. Follow-up measurements were scheduled 6 and/or 12 months later. Analysis of variance and regression models for repeated measures were used to estimate group and time effects. Serum Gd-IgA1 level was higher in initial samples for IgAN patients compared to those of healthy children (*P* < 0.0001). Serum Gd-IgA1 levels did not change over time for healthy controls but increased for IgAN patients (*P* = 0.001). Serum Gd-IgA1 level was elevated for 9 children with IgAN at study entry and remained elevated. Two of the 4 IgAN patients with initially normal Gd-IgA1 levels had a subsequent elevated level. The persistent elevation of the serum Gd-IgA1 level in children with IgAN enhances its utility as a potential diagnostic test for IgAN.

## 1. Introduction

IgA nephropathy (IgAN) is the most common type of chronic glomerulonephritis worldwide [1]. However, the true incidence of IgAN is difficult to determine because a renal biopsy is required for diagnosis. Diagnosis of IgAN may not be made in the milder cases or may be delayed until clinical manifestations are severe enough to necessitate this invasive procedure [2]. Development of a reliable serologic test for diagnosis of IgAN would be a major advance for early detection and treatment of this condition.

The pathogenesis of IgAN is related to aberrantly glycosylated IgA1 wherein some* O*-linked glycans in the hinge region contain terminal* N*-acetylgalactosamine (GalNAc) rather than a GalNAc-galactose structure [3]. The galactose-deficient IgA1 (Gd-IgA1) is recognized by anti-Gd-IgA1 autoantibodies [4]. This process results in formation of circulating immune complexes and then some complexes deposit in the glomerular mesangium, subsequently activating mesangial cells to proliferate and overproduce extracellular-matrix proteins and cytokines, thus inciting injury of the glomerulus [5–7].

In 2007, we found that serum levels of Gd-IgA1 were elevated in 75% of adult and pediatric patients with IgAN, suggesting that this measurement may be a useful diagnostic test for the condition [8, 9]. Subsequently, serum Gd-IgA1 level has been proposed as biomarker for IgAN [2, 10–12]. Serial levels of Gd-IgA1 have not previously been examined to determine if these levels remain stable over time in patients with IgA nephropathy or healthy controls. The only published data are for serial levels after kidney transplantation of adults with IgAN that showed that immunosuppressants, particularly corticosteroids, lowered serum Gd-IgA1 levels [11]. In addition, serum levels of Gd-IgA1 are significantly lower in children and adolescents as compared to adults [9] and African Americans have slightly lower levels than Caucasians [13]. The purpose of this study was to examine serial serum Gd-IgA1 levels over a period of 1 year in children with IgAN and healthy pediatric controls, to determine the effect of time on serum Gd-IgA1 level.

## 2. Materials and Methods

### 2.1. Ethical Considerations

The University of Tennessee Health Science Center Institutional Review Board approved the study protocol, “Non-Invasive Diagnosis of IgA Nephropathy” (IRB number 08-08771-XP). Informed written consent was obtained from each subject's legal guardian(s) and signed assent was obtained from all children 8 years of age or older.

### 2.2. Study Population

Children over 4 and less than 18 years of age were eligible for recruitment from the outpatient pediatric nephrology clinics and the Pediatric Clinical Research Unit at Le Bonheur Children's Hospital. Healthy controls were screened for renal disease by questionnaire at study entry and by urinalysis. Potential control subjects were excluded if more than trace blood or protein was detected on urine dipstick. The following demographic characteristics were obtained: date of birth, gender, and self-reported race/ethnicity. Subjects were excluded if they were a first-degree relative of previously entered patients or controls. Seventy-seven healthy controls were initially enrolled, but only 40 of them returned for follow-up visits.

The diagnosis of IgAN required renal biopsy showing IgA as the dominant or codominant immunoglobulin in a typical mesangial distribution in the absence of clinical and laboratory evidence for other systemic disease. No subject with IgAN was treated with a corticosteroid or nonsteroidal immunosuppressant during the study and no subject was studied during an episode of macroscopic hematuria. Children with IgA vasculitis (Henoch Schonlein purpura) were excluded.

### 2.3. Sample Handling

Blood and urine were obtained at study entry and at approximately 6 and 12 months later. The serum fractions of whole-blood specimens were frozen at −70°C and sent overnight in batches to the Department of Microbiology at the University of Alabama at Birmingham for determination of serum levels of Gd-IgA1 and total IgA. Samples were labelled by a unique code and the samples were analyzed in a blinded manner.

### 2.4. Serum Total IgA and Gd-IgA1 Levels

Serum levels of total IgA and Gd-IgA1 were determined by ELISA [8]. Serum total IgA was determined using IgA-calibrated human sera (Binding Site, Birmingham, UK) and the serum Gd-IgA1 was standardized to a polymeric Gd-IgA1 myeloma protein [8]. One unit/mL of Gd-IgA1 was equivalent to 1.0 *μ*g/ml of the Gd-IgA1 (Ale) standard. Total serum IgA levels are reported in *μ*g/ml.

### 2.5. Statistical Analysis

Depending on the underlying distribution, continuous variables are reported as a mean ± standard deviation or median (range) and categorical variables are reported as percentages. One-way analysis of variance (ANOVA) was used to compare mean serum levels of Gd-IgA1, total IgA, and percent Gd-IgA1/total IgA at baseline in patients with IgAN and healthy controls. To test the correlation and association between the outcome measures and age at baseline, we fit a simple linear regression model. Finally, we applied a repeated measure regression model with group, visit, and their interaction term using unstructured covariance structure, to test the group and visit marginal means and account for correlation in the data. This model made use of all available data points, regardless of the number of completed visits. Both ANOVA and regression methods incorporated Bonferroni adjustments for multiple comparisons between groups and visit time points. The 90th percentile for healthy controls was the cutoff point for elevated level of Gd-IgA1 as levels in patients were not normally distributed.

All statistical analyses were performed with SAS/STATv14.1 (SAS Institute Inc., Cary, NC, USA) and GraphPad Prism 6.0 (Graphpad Inc., San Diego, CA, USA). Statistical associations were considered significant at the alpha level of 0.05.

## 3. Results

Table 1 shows the demographic characteristics of both groups. The age of subjects in the healthy-control group versus the IgAN group did not significantly differ. African-American children comprised about two-thirds of the healthy controls, but only about one-third of the children with IgAN.

**Figure 1:**
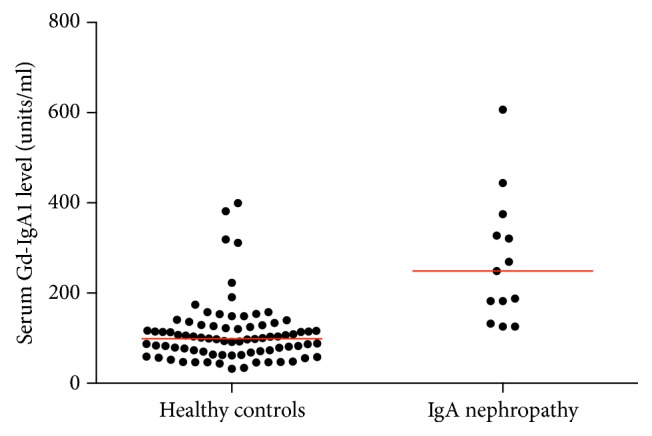
Serum Gd-IgA1 level at study baseline visit is shown for each study group. The red bars indicate median serum levels. Levels were significantly higher for the IgA nephropathy group as compared to the healthy-control group (*P* < 0.0001).

Data from the initial visit showed that children with IgAN had significantly elevated serum Gd-IgA1 levels compared to the healthy-control group (*P* < 0.0001) (Figure 1). The group of children with IgAN also had significantly higher serum total IgA levels as compared to the healthy-control group (*P* = 0.025) (Figure S1, supplemental appendix, in Supplementary Material, available online at https://doi.org/10.1155/2017/8210641). The percentage of Gd-IgA1 of total IgA was also higher in patients with IgAN than healthy controls (*P* < 0.0001) (Figure S2, supplemental appendix). Serum Gd-IgA1 level did not have a significant association with age at study baseline for healthy controls of both races (*P* = 0.36).

**Figure 2:**
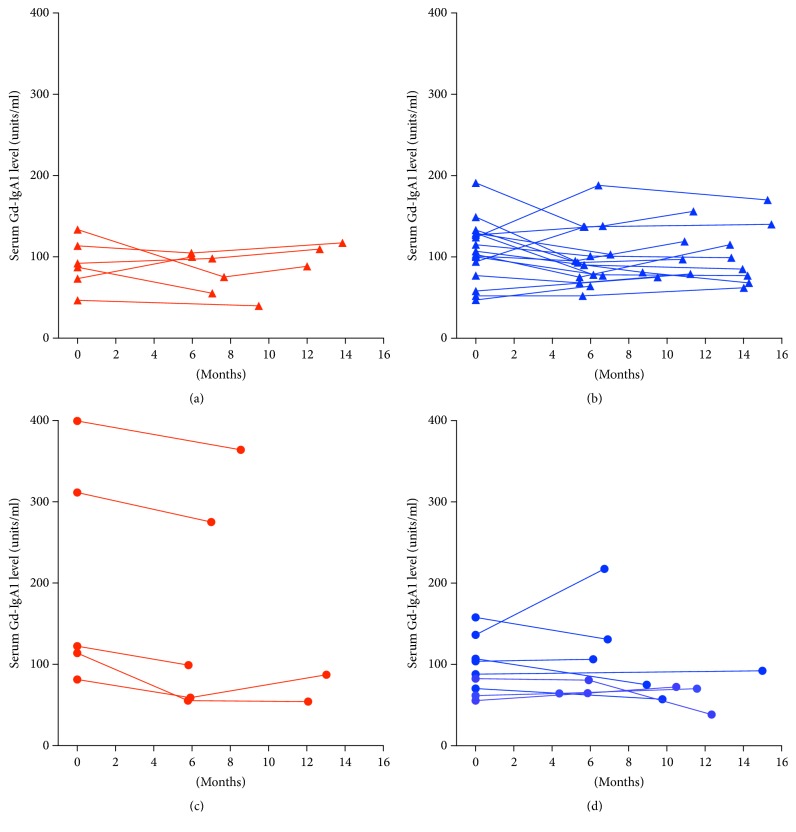
Spaghetti plots of serial serum Gd-IgA1 levels for healthy-control group are shown in Panel (a)—Caucasian males, Panel (b)—African-American males, Panel (c)—Caucasian females, and Panel (d)—African-American females.

**Figure 3:**
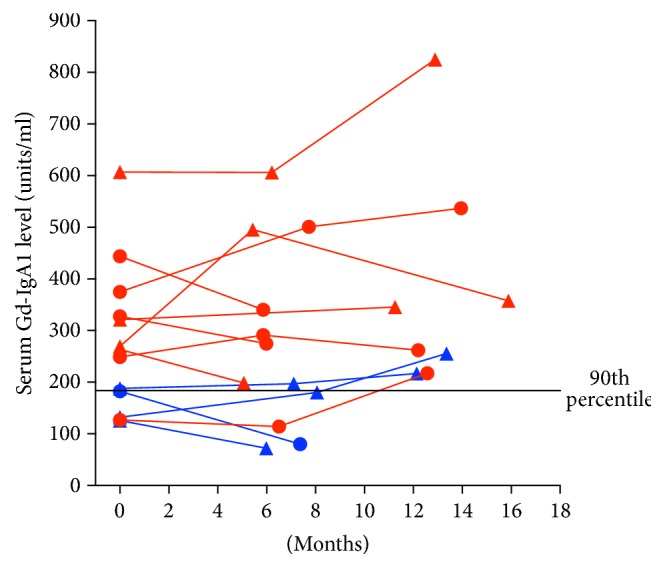
Spaghetti plots of serial Gd-IgA1 serum levels in pediatric patients with IgAN. Red symbols and lines represent Caucasian patients, while blue lines and symbols represent African Americans. Circles represent females and triangles represent males.

Serum Gd-IgA1 levels over the course of the study were plotted for each ethnicity-gender combination (Figure 2). The levels for each subject in these four groups appeared to be stable over the course of the study. Serum Gd-IgA1 level in the initial sample was above the 90th percentile for 9 of 13 (69%) subjects with IgAN (Figure 3). All 9 subjects with an elevated initial level had elevated levels in subsequent samples. Two of the 4 IgAN patients with an initially normal serum Gd-IgA1 level had an elevated level at 1-year of follow-up.

**Figure 4:**
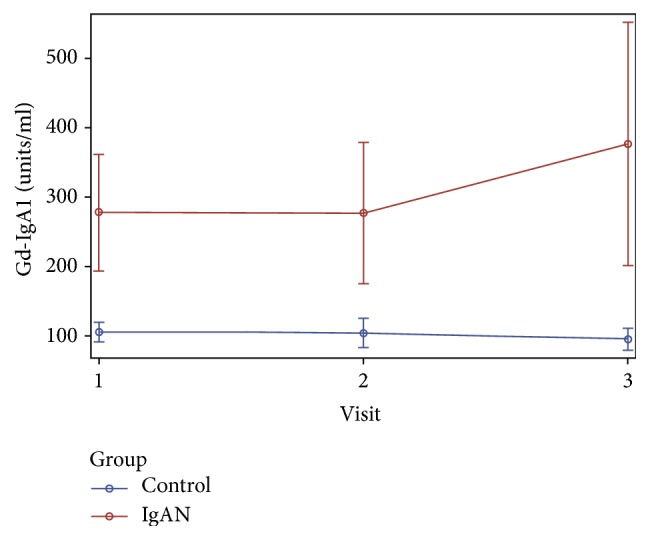
Mean and 95% confidence interval of serum Gd-IgA1 levels for each of the two study groups across three data collection points. The red line and circles represent IgAN patients and the blue line and circles represent healthy controls.

Figure 4 shows that, for the healthy-control group, the mean serum Gd-IgA1 levels did not significantly change over time (*P* = 0.13). For the children with IgAN, there was no significant difference between baseline and the 6-month follow-up measurement (*P* = 0.99); however, Gd-IgA1 levels at the 12-month follow-up were significantly higher than at the first 2 time points (*P* = 0.001 and *P* = 0.046, resp.).

## 4. Discussion

Our group previously demonstrated significantly elevated serum Gd-IgA1 levels in 22 pediatric patients with IgAN compared to 16 pediatric-control subjects [9]. No data from that previous study were included in the present study. This study further establishes that serum Gd-IgA1 levels are significantly elevated in pediatric patients with IgAN compared to levels in healthy-control pediatric subjects.

Serum IgA levels are known to increase during childhood and total serum IgA levels in young children are very low [14]. One study showed serum IgA levels in children to approach adult levels by the age of 6 to 7 years [14], although, in another study, adult levels for serum IgA were not achieved until the age of 16 years [15]. None of our healthy controls was younger than 6 years. If serum Gd-IgA1 levels parallel the total IgA levels for younger children, one must be careful in interpreting whether the Gd-IgA1 level is normal or elevated for children under age of 6 years.

Few studies have evaluated variation in serum total IgA levels using serial sampling. Veys et al. [16] showed that serum IgA levels fluctuate as much as 20% in samples collected 2 weeks apart, but changes over a month were not significant. Serum Gd-IgA1 levels showed little variation over 1 year for pediatric healthy-control subjects. However, almost half of the control subjects entered in the study did not return for serial measurements. Although the number of remaining healthy controls was more than 3 times higher than the number of IgAN patients in the study, it is possible that the noncomplying subjects differed somehow from the ones that continued in the study, introducing a source of bias for the results of our study.

Our study showed an increase in mean serum Gd-IgA1 levels at the 1-year visit in children with IgAN. This finding may be explained, in part, by the 4 IgAN patients with an initially normal serum Gd-IgA1 level. Two of those subjects returned for a 6-month but not for a 12-month blood draw, while for the other 2 the level increased at the final blood draw. Thus, the significant increase in Gd-IgA1 level might not be found in study with a larger sample size of subjects having levels for all 3 time points.

Although there appeared to be more variation in children with IgAN, those with an initially elevated level maintained an elevated level over the course of 1 year. Sustained elevations of serum Gd-IgA1 levels over time for most children with IgAN contributes to the utility of this biomarker as a potential diagnostic test.

## Supplementary Material

Figure S1: Total serum IgA level for children with IgA nephropathy and healthy-controls.
Figure S2: Percentage of serum Gd-IgA1/total serum IgA for children with IgA nephropathy and healthy-controls.

## Figures and Tables

**Table 1:** Demographic characteristics for subject groups.

Group	Number	Males	Caucasians	Age at study entry median (range) years
Healthy controls	77	48 (62%)	32 (41%)	11.7 (6.8–17.6)
Patients with IgAN	13	6 (46%)	9 (69%)	14.3 (8.6–17.2)

## References

[1] D'Amico G G. (1987). The commonest glomerulonephritis in the world: IgA nephropathy. *Quarterly Journal of Medicine*.

[2] Wyatt R. J., Julian B. A. (2013). Medical progress: IgA nephropathy. *The New England Journal of Medicine*.

[3] Tomana M., Matousovic K., Julian B. A., Radl J., Konecny K., Mestecky J. (1997). Galactose-deficient IgA1 in sera of IgA nephropathy patients is present in complexes with IgG. *Kidney International*.

[4] Suzuki H., Fan R., Zhang Z. (2009). Aberrantly glycosylated IgA1 in IgA nephropathy patients is recognized by IgG antibodies with restricted heterogeneity. *The Journal of Clinical Investigation*.

[5] Ebefors K., Liu P., Lassén E. (2016). Mesangial cells from patients with IgA nephropathy have increased susceptibility to galactose-deficient IgA1. *BMC Nephrology*.

[6] Seki G., Tanaka M., Someya T., Nagata M., Fujita T. (2011). Aberrantly glycosylated IgA1 as a factor in the pathogenesis of IgA nephropathy. *Clinical and Developmental Immunology*.

[7] Suzuki H., Kiryluk K., Novak J. (2011). The pathophysiology of IgA nephropathy. *Journal of the American Society of Nephrology*.

[8] Moldoveanu Z., Wyatt R. J., Lee J. Y. (2007). Patients with IgA nephropathy have increased serum galactose-deficient IgA1 levels. *Kidney International*.

[9] Lau K. K., Wyatt R. J., Moldoveanu Z. (2007). Serum levels of galactose-deficient IgA in children with IgA nephropathy and Henoch-Schönlein purpura. *Pediatric Nephrology*.

[10] Sun Q., Zhang Z., Zhang H., Liu X. (2016). Aberrant IgA1 glycosylation in IgA nephropathy: A systematic review. *PLoS ONE*.

[11] Kim M. J., Schaub S., Molyneux K., Koller M. T., Stampf S., Barratt J. (2016). Effect of immunosuppressive drugs on the changes of serum galactose-deficient IgA1 in patients with IgA nephropathy. *PLoS ONE*.

[12] Coppo R. (2017). Biomarkers and targeted new therapies for IgA nephropathy. *Pediatric Nephrology*.

[13] Hastings M. C., Moldoveanu Z., Julian B. A. (2010). Galactose-deficient IgA1 in African Americans with IgA nephropathy: serum levels and heritability. *Clinical Journal of the American Society of Nephrology*.

[14] Buckley R. H., Dees S. C., O'Fallon W. M. (1968). Serum immunoglobulins. I. Levels in normal children and in uncomplicated childhood allergy. *Pediatrics*.

[15] Stiehm E. R., Fudenberg H. H. (1966). Serum levels of immune globulins in health and disease: a survey. *Pediatrics*.

[16] Veys E. M., Wieme R. J., Van Egmond J., Gabriel P., Van Der Jeught J. (1977). Short term variation of human immunoglobulin levels with an estimation of the day to day physiological variability. *Clinica Chimica Acta*.

