# Supplementary material for: Serial Galactose-Deficient IgA1 Levels in Children with IgA Nephropathy and Healthy Controls
Source: Int J Nephrol. 2017 Nov 26;2017:8210641. doi: 10.1155/2017/8210641 (PMC5733148; doi:10.1155/2017/8210641)
Supplement: Supplementary file 1 — Figure S1: Total serum IgA level for children with IgA nephropathy and healthy-controls. Figure S2: Percentage of serum Gd-IgA1/total serum IgA for children with IgA nephropathy and healthy-controls. [file 8210641.f1.docx]

**Supplemental Appendix**

**Serial Galactose-Deficient IgA1 Levels in Children with IgA Nephropathy and Control Populations**

**John T. Sanders,^1^ M. Colleen Hastings,^2,3^ Zina Moldoveanu,^4^ Jan Novak,^4^ Bruce A. Julian,^4^ Zoran Bursac,^2^ Robert J. Wyatt^2,3^**

^1^ Sanford Children’s Hospital, Sioux Falls, SD 57117, USA

^2^ University of Tennessee Health Sciences Center, Memphis, TN 38013, USA

^3^ Children’s Foundation Research Institute, Memphis, TN 38013, USA

^4^ University of Alabama at Birmingham, Birmingham, AL 35294, USA

Correspondence should be addressed to Robert J. Wyatt; [rwyatt@uthsc.edu](mailto:rwyatt@uthsc.edu)

**Figure S1:** Serum total IgA levels at baseline visit for the two study groups. The red bars indicate median levels. The group of children with IgA nephropathy had higher levels as compared with the healthy-control group (*P* = 0.025).

**Figure S2:** The percentage of Gd-IgA1 of total IgA was also higher in the group of children with IgA nephropathy as compared with the healthy-control group (*P* < 0.0001).
